# Depression and Anxiety in 336 Elective Orthopedic Patients

**DOI:** 10.3390/jcm13237354

**Published:** 2024-12-03

**Authors:** Leszek Kuik, Piotr Łuczkiewicz

**Affiliations:** 1 Clinical Department of Orthopaedics, Kinetic Organ Traumatology and Hand Surgery, University Clinical Center in Gdańsk, 80-952 Gdańsk, Poland; 2 II Clinic of Orthopaedics and Kinetic Organ Traumatology, Gdańsk Medical University, 80-384 Gdańsk, Poland

**Keywords:** depression, anxiety, orthopaedics, psychiatry, SSRI

## Abstract

**Background/Objectives**: Depression and anxiety are the two most common mental health disorders that can affect the well-being of the entire body. Multiple studies confirm that they can threaten the musculoskeletal system and the effects of orthopedic treatment as well. In turn, orthopedic disorders may worsen the symptoms of depression and anxiety. The study is aimed at assessing the incidence of depressive and anxiety disorders in orthopedic patients of our department and what are the characteristics of orthopedic patients regarding depressive disorders. **Methods**: After obtaining personal consent for trial, 336 patients undergoing elective orthopedic surgery over a 12-month period were evaluated. Preoperatively, patients completed surveys containing questions from the PHQ-9 and GAD-7 forms. The pain was assessed with the VAS scale of 0–10 points and the information on the current psychiatric treatment was acquired. Patients were divided into subgroupsand statistical analysis was performed. **Results**: The incidence of moderate depression and generalized anxiety symptoms in orthopedic patients was 12.2% and 11.3%, respectively (several times higher than in the general population). In the group most at risk of depression, i.e., women over 40 and with foot and ankle diseases, the incidence of treated depression was 36%. In foot and ankle patients, prevalence for depression was more than three times higher (OR = 3.24, 95% CI 1.542–7.24) compared to the reference group. **Conclusions**: The problem of depression and generalized anxiety in orthopedic patients is clearly more common than in the general population. In our study, patients with foot and ankle disorders are the most vulnerable to depression.

## 1. Introduction

Depression and anxiety are the most common mental health disorders that affect people globally. Long-term depression occurs in approximately 5% of the world population, while generalized anxiety occurs in approximately 3–4%. Shorter episodes of the above-mentioned disorders occur in several times higher percentages of people [1,2,3].

Patients who struggle with degenerative diseases of the joints and tendons, and the resulting challenges of pain and mobility impairment, are particularly susceptible to the co-occurrence of depression and anxiety [4]. Injury to the musculoskeletal system is an important factor influencing the level of stress, as well as the severity of depressive disorders [5,6,7].

Symptoms of depression, in turn, may significantly affect the deterioration of functional results and satisfaction in patients after surgical treatment, although the research results are not clear [8,9,10,11,12,13]. Yet, from another point of view, the health of the musculoskeletal system and activity is a known factor that reduces stress and symptoms of depression [14,15].

For the abovementioned reasons, in the practice of both orthopedic departments and clinics, awareness of the occurrence of depression and generalized anxiety disorder in patients is crucial for optimizing the results and cost-effectiveness of treatment. Despite the universality of the problem, the number of studies describing the frequency and factors influencing the mental state of patients with musculoskeletal disorders seems to be insufficient.

The study aims to provide information on the incidence of depression and anxiety in orthopedic patients who were admitted for elective surgery and what are the characteristics of orthopedic patients regarding depressive disorders.

## 2. Materials and Methods

The study was performed over a 12-month period and, primarily, 523 patients were included for the study. One hundred eighty-seven patients were excluded due to lack of consent or inappropriate completion of the forms. Finally, 336 patients, who were electively admitted and operated on at the Orthopedic Department of University Clinical Centre in Gdańsk, were included in the study. As the study involved just retrospective medical data of commonly used surveys, there was no requirement for the consent of the local Ethical Committee.

Patients with multiple organ injuries and severe trauma were excluded from the study due to the possibility of obtaining inadequate answers caused by a sudden and significant change in their life situation. Patients with dementia or mental disability that prevented them from completing the survey on their own were also excluded, as well as those who refused to undergo the examination. Patients were not assisted in filling the questionnaires as they were designed for self-completion. The assessment involved the results of commonly recognized screening forms for depression (Patient Health Questionnaire—PHQ-9) and generalized anxiety disorder (GAD-7) [3,16,17,18]. The surveys also included questions about depression treatment, type of medications taken, and the level of pain determined by visual analogue scale (VAS) and body mass index (BMI), as well as site of operation, distinguishing foot and ankle (F&A), lower leg, knee, thigh, hip, pelvis, shoulder, arm, elbow, forearm, and hand and wrist (H&W).

The PHQ-9 form consists of nine questions that determine the severity of depressive symptoms over the last 2 weeks, with a score from 0 to 3 points each. A total score of 10 or more allows for the diagnosis of a moderate depression episode, from 15 to 19 points—moderately severe, and 20 or more indicates severe depression episode. In some studies, with a score of five to nine points, a mild depression episode is also distinguished. The GAD-7 form contains seven questions that assess the severity of anxiety symptoms on a scale from zero to three points each. A total score of 10 or more indicates features of generalized anxiety disorder. If at least 10 points were obtained on any of the forms, patients were informed about the indications for consulting with a mental health or psychology specialist. The surveys also included pain assessment with VAS from 0 to 10 points.

In statistical analysis, the one-dimensional ANOVA was performed to verify the hypothesis that there are statistically significant differences in mean age and BMI between the body area subgroups. Kruskal–Wallis rank sum test was used for VAS pain score. The post-hoc Tukey’s test was performed to compare all pairs of groups to identify which specific groups differed significantly from each other. Fisher’s exact test was used to check the relationship between the operated body area and the gender predominance. To find independent risk factors at each point, stepwise estimation of logistic or linear regression models (based on the type of dependent variable) was performed. Additionally, 3-fold cross-validation was used on the training set. The explanatory variables were selected from the following variables: gender (F/M), BMI, body region, and age range (<20, 20–29, 30–39, 40–49, 50–59, 60–69, ≥70). Of all the estimated models, the best ones were selected based on the AIC criterion (the lower its value, the “better” the model). It shows the results of the estimation of the logistic regression model, in which the dependent variable is depression (taking the values yes—1/no—0). The model considers variable gender and body area. The reference value for age is people <20 years of age, and for body region, the reference value is shoulder. The analysis of covariance was performed between PHQ-9, GAD-7, and VAS scores.

## 3. Results

**Figure 1:**
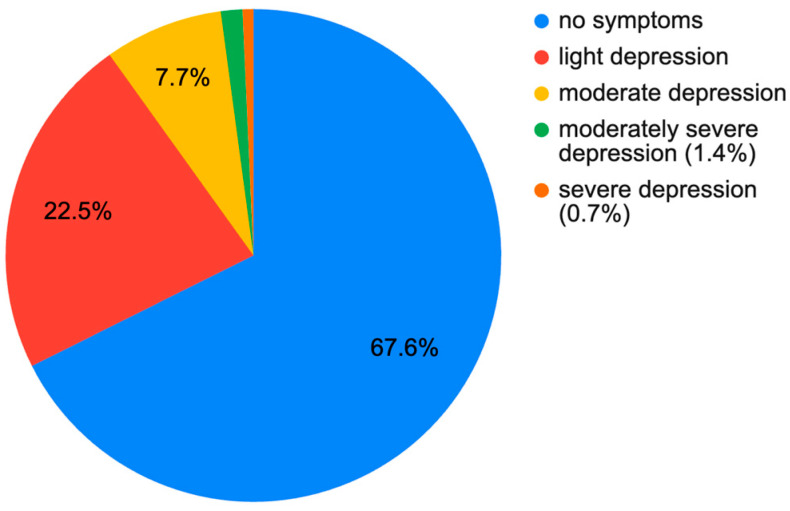
Level of depression in the group of orthopedic patients WITHOUT pharmacological treatment of depression according to PHQ-9.

**Figure 2:**
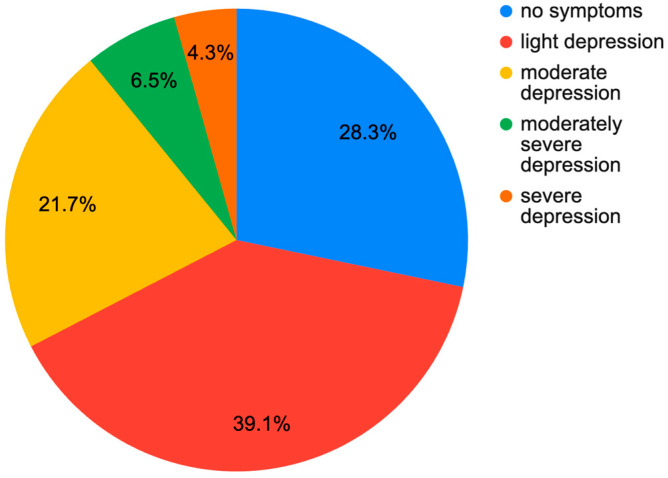
Level of depression in the group of orthopedic patients WITH pharmacological treatment of depression according to PHQ-9.

Among the 336 patients examined, there were 193 female (57%) and 143 male (43%). The median age in the entire group was 56 years (41.0, 67.0). The median BMI was 27.1 (24.0, 30.3). The median PHQ-9 score was 3.0 (1.0, 6.0). The median VAS score was 4.00 (2.75, 7.00). The median GAD-7 score was 3.0 (1.0, 6.0). A result indicating active symptoms of mild depression was observed in 87 people (25.9%). Moderate depression was found in 32 people (9.2%). Results for moderately severe and severe depression were found in 11 people (3.0%). Symptoms of generalized anxiety were observed in 38 people (11.3%). Current treatment for depression was reported in 37 women (19%) and 9 men (6%), which provides a total of 13.7% of the entire group. In the group not treated pharmacologically, symptoms of at least mild depression were present in 31.7% of patients. At least moderate depression was present in 10.4%. In the pharmacologically treated group, no symptoms of depression were present in only 28.3%, while symptoms of at least moderate depression were present in 32.3% of patients. The exact distribution is shown in Figure 1 and Figure 2.

Among patients treated for depression, 6 (13%) patients were treated with medications only in the case of symptoms, 27 (58.7%) were constantly treated with one drug, 10 (21.7%) were treated with 2 drugs, and 3 (6.5%) were treated with 3 drugs. The most commonly used drugs were Serraline and Trazodone (in eight cases each), Escitalopram (six cases), Pregabalin and Quetiapine (five cases each), and Duloxetine and Venlafaxine (four cases each).

**Figure 3:**
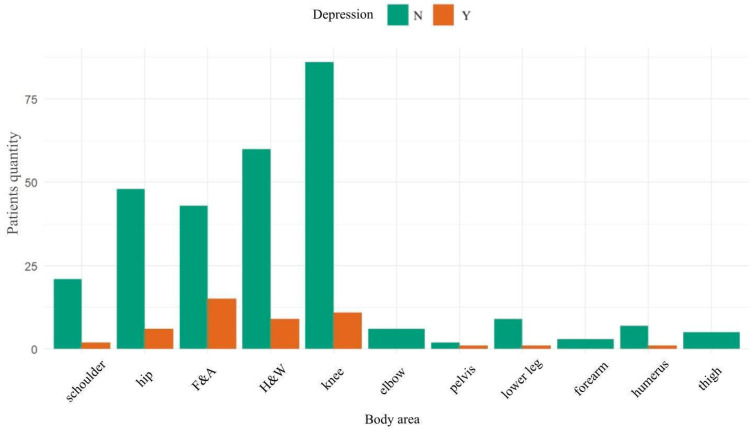
Distribution of patients with and without depression treatment according to operated body area.

The groups of patients with and without treated depression did not differ significantly in terms of age and BMI. However, noticeable differences appeared in the incidence of depression in subgroups of patients according to the area of the body operated on, as shown in Figure 3. The highest percentage of patients treated for depression was in the foot and ankle patient subgroup.

Subsequently, the analysis of the influence of independent factors on the occurrence of symptoms and treatment of depression was performed, paying attention to the operated area of the body. The quantitative results are presented in Table 1.

Significant differences in age were found between groups of patients with diseases of various body areas (*p* < 0.01). It showed that a subgroup of patients with hip diseases was older than F&A patients and knee patients, just with slight significance (*p* < 0.03 and <0.06, respectively). 

Significant differences in BMI were found between groups of patients with diseases to various parts of the body (*p* = 0.02). However, post-hoc tests did not show any significant differences between specific groups.

There was no significant relationship between the operated body area and the gender predominance (*p* = 0.3). No significant differences were found in the results of GAD-7 and PHQ-9 tests between body area groups.

**Figure 4:**
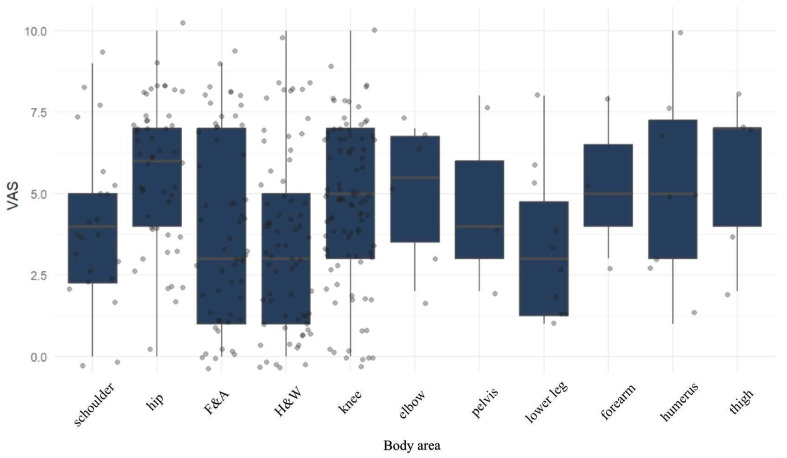
Level of pain (VAS) in patients according to operated body area.

Significant differences in pain VAS scores were found between groups of patients with injuries to various parts of the body (*p* = 0.004). Post-hoc tests showed that the median pain VAS in patients with hip disorders is statistically significantly higher compared to H&W patients (6 (4–7) vs. 3 (1–5), *p* = 0.0005) and F&A patients (6 (4–7) vs. 3 (1–7), *p* = 0.0277). The detailed distribution is shown in Figure 4.

As for independent risk factors, including age, body area, and gender groups, only the lowest *p*-values and their parameters are presented in Table 2. The F&A area surprisingly had a stronger coincidence with depression treatment than the female gender. These model parameters, except age, are statistically significant (*p* < 0.05).

The accuracy of the model on the test set was 82.5%, with a sensitivity of 0.18 and a specificity of 0.93. This model is statistically significantly better than the model with one independent variable (LRT test *p*-value < 0.05)

Considering the OR value, it can be concluded that male patients have a 63% lower risk of depression compared to female patients. It was also shown that F&A patients have a more-than-three-times-higher risk of depression compared to patients from the reference group.

**Figure 5:**
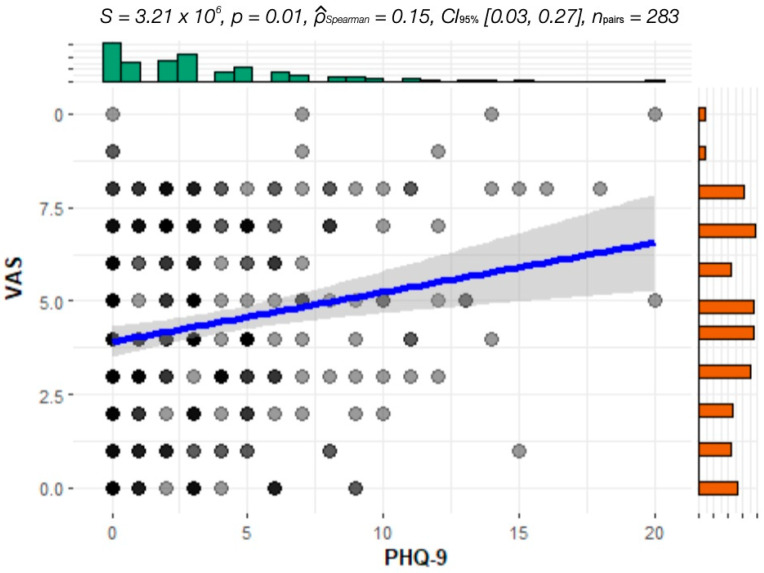
Covariance of VAS and PHQ-9 of patients WITHOUT treatment of depression.

**Figure 6:**
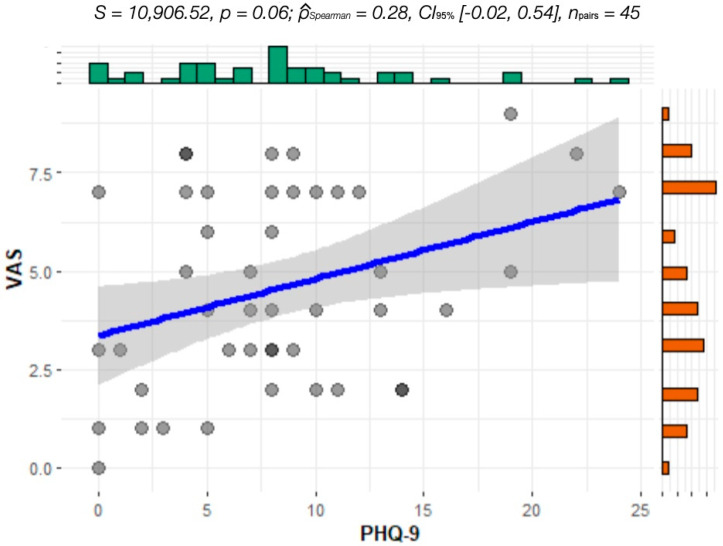
Covariance of VAS and PHQ-9 of patients WITH treatment of depression.

The analysis of covariance performed between VAS and PHQ-9 levels showed a positive correlation (*p* = 0.0017), which is visualized in Figure 5 and Figure 6. There was no significant difference in the correlation between patients with and without depression treatment. Analysis of VAS versus anxiety level (GAD-7) did not reveal any correlation.

The incidence of depression in orthopedic patients is 13.7% in our population. Even in patients that were never treated for depression, at least mild depression was present in 31.7% of cases. Our observations indicate that depression treatment usually reduces depression symptoms by one level according to the PHQ-9, and only 1/3 of patients show no symptoms.

Based on the OR, male patients have a 63% lower risk of depression compared to female patients. It was also shown that F&A patients have a more-than-three-times-higher odds ratio of depression treatment compared to patients from the reference group. The incidence of depression was especially high in women over 40 years old and affected 36% of this group.

Some contradiction is noticeable in the comparison of groups of patients with hip and F&A complaints. The first of these groups is significantly older, reporting the greatest pain, and, at the same time, has the lowest incidence of depression, while the F&A group, despite average pain and age, has a much higher incidence of depression treatment. 

## 4. Discussion

According to the WHO report, depression affects approximately 5% of the population globally [2]. Javal et al., analyzing a recent Global Burden of Disease dataset, estimated the prevalence of depression and anxiety disorders at 2–3% [19]. In the report of the Polish National Health Fund (NFZ) from the period 2013–2023, the numbers concerning depression treatment correspond to approximately 4% of the population [20]. All of the above studies indicate a significantly lower incidence of depression than in our study involving only orthopedic patients.

Muscatelli et al., in the review work on orthopedic trauma, depression, and posttraumatic stress disorder, revealed that the depression prevalence in patients after orthopedic trauma reaches 32.6% after 6 months and 16% after 2 years post-incident [6]. Weekes et al. described that 24% of patients had major depressive disorder at 1 year after the elective operation of the shoulder, while preoperatively, the numbers corresponded to over 50% of the group [7]. McQuillan et al. published similar results with approximately ¼ of patients with diagnosed depression [10].

The abovementioned numbers are consistent with our results, which showed that in orthopedic patients, 38.7% of patients showed symptoms of at least mild depression, 12.2% of moderate or severe depression, and 11.3% showed generalized anxiety symptoms. As much as 13.7% of patients reported being currently treated for depression. That shows the incidence of generalized anxiety and depression in orthopedic patients several times greater than in the whole population.

Considering the operated area of the body, a surprisingly higher rate of treated depression was found in patients with foot and ankle disorders, especially among women over 40. The condition occurred approximately in 36%, i.e., three times more often than in the reference group of orthopedic patients and several times more than in the general population. Although F&A patients in our study did not show higher scores on the PHQ-9 and GAD-7 tests, this may be due to the appearance of successfully treated depression. 

The U.S. National Center for Health Statistics study, which includes an assessment of depression in different age groups, showed the lowest level observed in the age group of 30 to 44 years [21]. Similarly, according to the report of the Polish National Health Fund (NFZ), the age group where the therapy was most frequent was 65–74 years [19]. The abovementioned results are not consistent partially with our observation for F&A patients who were significantly more often treated for depression than patients with hip joint problems, despite their lower VAS pain score and relatively lower age.

Converging results for F&A patients were obtained by Nakagawa et al., where the prevalence of anxiety and depression was 30% and 27%, respectively [22]. Also, in the work by Awale et al., a strong correlation between foot pain and depression was observed [23].

Further associations of depression and foot diseases are represented by numerous studies on diabetic foot and related non-healing ulcers [24,25,26].

Henry et al. described that patients with foot and ankle diseases and with depression have increased expectations regarding the treatment, less postoperative functional improvement, and less satisfaction. However, the above work does not mention possible differences resulting from the operated body area [9].

Considering the above reports, it can be inferred that orthopaedic patients, and especially F&A patients, may require a more sensitive and gentle approach. Appropriate patient selection, proper setting of expectations, properly selected treatment of foot diseases, and appropriately directed rehabilitation of the foot and ankle joint may have a beneficial impact not only on general fitness but also on the patient’s mental well-being. Regarding rehabilitation as well as non-pharmacological treatment, there is considerable support in the literature for the beneficial effect of foot reflexology on symptoms of depression. This also indicates how important individual orthopedic insoles are [27,28].

As Trivedi described in his review, pain and depression share neurological pathways, and thus physical and mental symptoms are commonly related. This explains the increased incidence of symptoms of depression in orthopedic patients presenting higher pain yet does not explain such a significant difference in the frequency of depression in patients with foot and ankle diseases [29].

The neuroanatomical basis of observed differences certainly requires further research.

So far, in the study by Vajapey et al., it was observed that the patient’s depression symptoms improved after orthopedic operative intervention [13]. However, there has been no clear confirmation that pharmacological treatment of depression significantly affects the results of surgical treatment of orthopedic diseases [30].

Nonetheless, it is clear that depression negatively affects the functional results of surgical treatment of patients, increases infections and readmission rates, worsens the results of rehabilitation, and is associated with an increased total cost of treatment [7,13,30].

It is safe to assume that the problem of depression in orthopedic patients is not marginal. Unfortunately, orthopedic surgeons do not have sufficient scientific support to assist them in the decision-making process in case of a depressive patient. Studies are few, non-complementary, and do not provide simple action plans. As with patients with foot and ankle conditions, there are isolated studies, such as Henry et al.’s, that only provide an estimate of the prevalence of the problem, not a solution. They also do not emphasize the differences in the orthopedic patient population and the special needs of specific patient groups.

In orthopedic practice, patients who are less compliant and less susceptible to treatment are regularly encountered. There is a high probability that such a patient suffers from depression or generalized anxiety regardless of being aware of it. Of course, it is not the role of an orthopedist to conduct diagnostics or treatment of mental disorders. However, making both the doctor and the patients aware of these conditions and referring the latter to an appropriate specialist can significantly improve cooperation in the treatment of musculoskeletal disorders.

Antidepressants and anxiolytics are used in the treatment of chronic pain in diseases, such as cancer, where the prevalence of anxiety and depression is also very high. However, as an optional adjuvant pain therapy, these drugs can be administered from the first step of the analgesic ladder, together with simple nonsteroidal anti-inflammatory drugs [31,32].

Perhaps in cases of milder depression, associated with significant functional impairment or chronic pain of the motor organ, it would be justified to use safe adjuvant drugs. Tianeptine or Trazodone could play a more meaningful role at the level of orthopedic outpatient clinics. However, this requires confirmation in further research.

Our study has several limitations. First of all, our group of 336 patients was relatively small for population studies. It included approximately 2/3 of elective surgery patients due to many refusals to participate in the study or incorrect completion of questionnaires. We believe it does not influence our results greatly as some patients did not consent to the study because they did not see the problem of depression in themselves, and others did not do so because of shame or fear of exclusion. Nonetheless, the study covers patients from the entire year, which excludes potential seasonal fluctuations of the results. The single-center nature also reduces the power of this study.

Treatment for depression criteria only involved taking any antidepressant medication. The total number or type of medications, duration of therapy, and the possible occurrence of psychotherapy were not considered. The division into body regions was also rather conventional, while the foot and ankle group and hand and wrist group were combined due to the organizational method of the department’s work.

## 5. Conclusions

Awareness of a patient’s depression and anxiety should be considered an integral part of every doctor’s practice. It is also a significant problem in orthopedic practice and has an impact on the progress of treatment of musculoskeletal diseases. The incidence of depression and anxiety in orthopedic patients is clearly higher than in the average population. Physical exercises, exceptionally dependent on motor skills, are a recognized element of the treatment of depressive disorders. The clearly increased incidence of treated depression observed in people suffering from foot and ankle disorders provides a basis for further neuroanatomical research and the development of optimal care for orthopedic patients with depression, as well as psychiatric patients with motor organ impairment.

## Figures and Tables

**Table 1:** Quantitative results of basic parameters in subgroups, depending on the operated body area. Only the groups exceeding 20 patients are displayed.

Parameter	Total N = 336	F&A N = 58	H&W N = 69	Shoulder N = 23	Hip N = 54	Knee N = 97
**Age ^1^**	56.0 (41.0, 67.0)	57.0 (42.0, 63.8)	51.0 (41.0, 65.0)	55.0 (40.5, 61.0)	**65.5** **(55.3, 71.0)**	55.0 (38.0, 69.0)
**Female**	193 (57.4%)	40 (69.0%)	42 (60.9%)	9 (39.1%)	25 (46.3%)	54 (55.7%)
**Male**	143 (42.6%)	18 (31.0%)	27 (39.1%)	14 (60.9%)	29 (53.7%)	43 (44.3%)
**BMI ^1,2^**	27.1 (24.0, 30.3)	26.5 (23.2, 29.1)	25.5 (23.2, 28.8)	26.5 (22.5, 29.1)	27.4 (24.2, 30.5)	27.8 (25.6, 31.8)
**Treated depression (N)**	46 (13.7%)	**15** **(25.9%)**	9 (13.0%)	2 (8.7%)	6 (11.1%)	11 (11.3%)
**PHQ-9 ^1^**	3.0 (1.0, 6.0)	3.0 (1.3, 7.0)	3.0 (1.0, 7.0)	4.0 (2.0, 6.5)	3.0 (0.3, 6.8)	3.0 (1.0, 6.0)
**VAS ^1^**	4.0 (2.8, 7.0)	3.0 (1.0, 7.0)	3.0 (1.0, 5.0)	4.0 (2.3, 5.0)	**6.0** **(4.0, 7.0)**	5.0 (3.0, 7.0)
**GAD-7 ^1^**	3.0 (1.0, 6.0)	3.0 (0.0, 6.0)	3.0 (1.0, 5.0)	3.0 (1.5, 5.5)	3.0 (1.0, 6.0)	2.0 (0.0, 4.3)

^1^ median (Q1,Q3); ^2^ (kg/m^2^). Significantly different values are marked in bold.

**Table 2:** Results of estimation of the logistic regression model with the depression treatment Y/N as the explained variable.

Characteristic	OR	95% CI ^1^	*p*-Value
Age: 20–29	0.23	0.01, 1.24	0.2
Gender: Male	0.37	0.15, 0.83	**0.021**
Body area: F&A	3.24	1.42, 7.24	**0.005**

^1^ OR = Odds Ratio, CI = Confidence Interval. Statistically significant values are marked in bold.

## Data Availability

Data are available through direct contact with the corresponding author.
